# Supplementary material for: Maternal Diet May Modulate Breast Milk Microbiota—A Case Study in a Group of Colombian Women
Source: Microorganisms. 2023 Jul 14;11(7):1812. doi: 10.3390/microorganisms11071812 (PMC10384792; doi:10.3390/microorganisms11071812)
Supplement: Supplementary file 1 [file microorganisms-11-01812-s001.zip › Figure S2. Rarefaction curves..pdf]

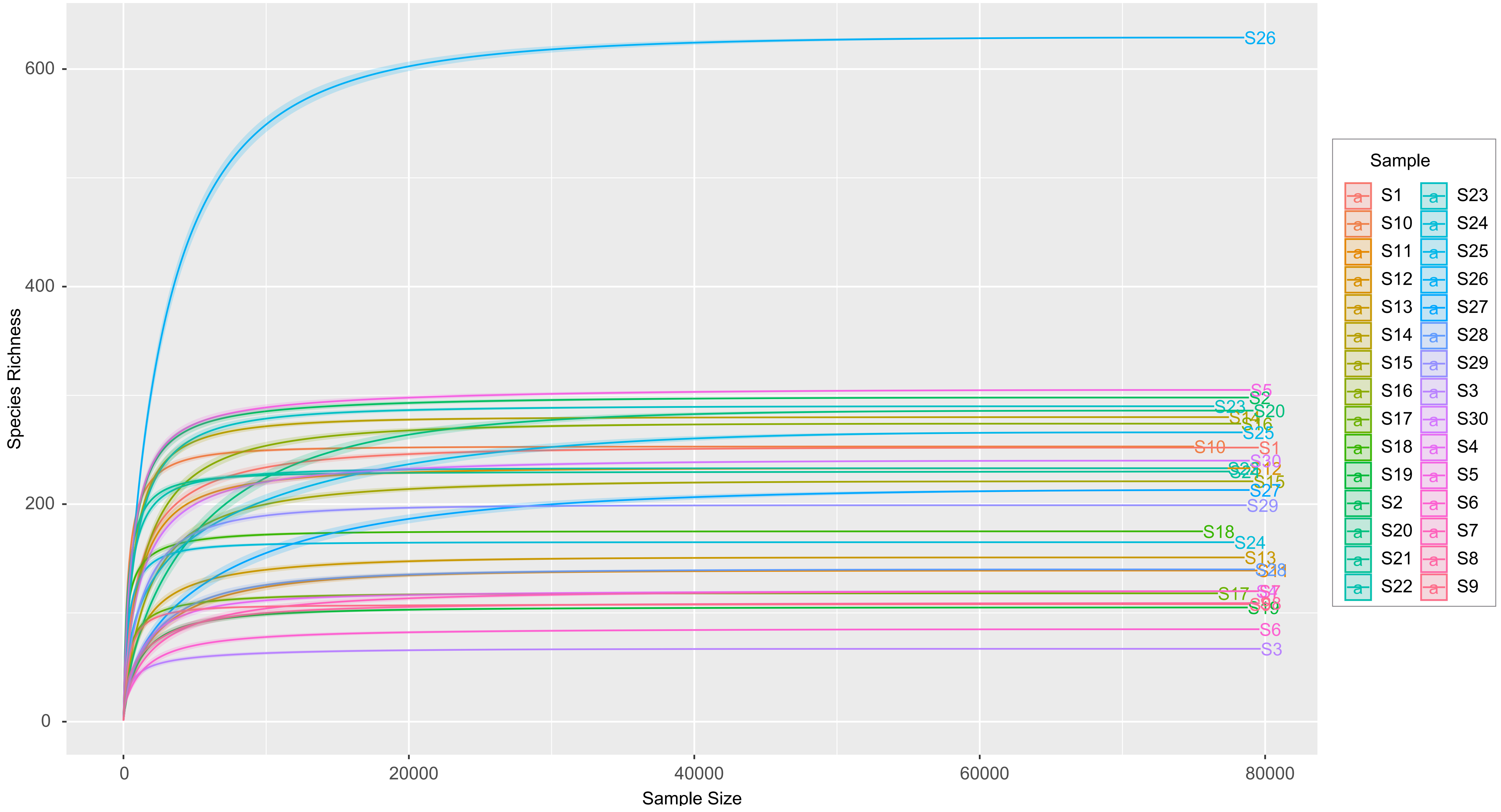
Species Richness
Sample Size
0
200
400
600
0
20000
40000
60000
80000
S26
S5
S2
S23
S20
S25
S10
S1
S30
S12
S18
S24
S13
S27
S29
S17
S19
S6
S3
Sample
S1
S10
S11
S12
S13
S14
S15
S16
S17
S18
S19
S2
S20
S21
S22
S23
S24
S25
S26
S27
S28
S29
S3
S30
S4
S5
S6
S7
S8
S9
