# Supplementary material for: Maternal Diet May Modulate Breast Milk Microbiota—A Case Study in a Group of Colombian Women
Source: Microorganisms. 2023 Jul 14;11(7):1812. doi: 10.3390/microorganisms11071812 (PMC10384792; doi:10.3390/microorganisms11071812)
Supplement: Supplementary file 1 [file microorganisms-11-01812-s001.zip › Table S1. Concordance test to assess reproducibility between 24-.pdf]

Table S1. Concordance test to assess reproducibility between 24-hour recall and Food Consumption Frequency of a group of pregnant women (n = 7).

**Nutrients**	**Adjusted R24h**<br><br>**Me (MAD)**	**Frecuency**<br><br>**Me (MAD)**	**Wilcoxon p**	**Hodges - Lehmann**	**95% CI**	**Biserial Rank Correlation Coefficient (95% CI)**	**Concordance Correlation Coefficient (95% CI)**
Calories (Kcal)		3122 (1407)	0.156*	-1145.430	-0.920, 0.065	-0.453 (-0.740, 0.101)	0.052 (-0.025, 0.128)
Protein (g)		122.9 (23.4)	0.109*	-54.737	-0.938, -0.068	-0.507 (-0.766, 0.034)	-0.012 (-0.113, 0.089)
Total Fat (g)	61.8 (4.5)	76.6 (44.0)	0.297*	-30.108	-0.880, 0.272	-0.342 (-0.685, 0.222)	0.059 (-0.070, 0.186)
Saturated (g)	25.50 (0.27)	32.80 (21.90)	0.375*	-10.835	-0.858, 0.354	-0.325 (-0.677, 0.238)	0.008 (-0.020, 0.036)
Monounsaturated (g)	22.00 (1.70)	26.10 (20.80)	0.4069*	-11.440	-0.834, 0.426	-0.330 (-0.679, 0.233)	0.020 (-0.178, 0.216)
Polyunsaturated (g)	12.50 (1.90)	15.10 (7.80)	0.578*	-2.647	-0.808, 0.489	-0.211 (-0.617, 0.339)	0.259 (-0.024, 0.504)
Total Carbohydrate (g)		400.0 (127.3)	0.156*	-139.523	-0.920, 0.065	-0.426 (-0.727, 0.133)	0.025 (-0.012, 0.062)
Dietary fiber (g)		34.4 (12.0)	0.078*	-19.227	-0.955, -0.228	-0.543 (-0.782, -0.015)	-0.388 (-0.128, 0.051)
Calcium (mg)		1741 (882)	0.297*	-551.357	-0,880, 0.272	-0.341 (-0.685, 0.222)	0.086 (-0.329, 0.474)

Iron (mg)	32.3 (23.4)	0.578*	-25.360	-0.808, 0.489	-0.316 (-0.672, 0.246)	0.153 (-0.161, 0.438)
Potassium (mg)	5458 (760)	0.047	- 3,034.532	-0,971, - 0.425	-0.540 (-0.781, - 0.010)	0.046 (-0.023, 0.134)
Magnesium (mg)	460.0 (85.9)	0.078*	-232.938	-0.955, 0.228	-0.542 (-0.782, - 0.013)	0.025 (-0.068, 0.117)
Zinc (mg)	18.6 (2.7)	0.078*	-9.828	-0.955, 0.228	-0.583 (-0.801, - 0.072)	-0.006 (-0.037, 0.025)
Manganese (mg)	4.8 (1.7)	0.078*	-1.755	- 0.955. - 0.228	-0.479 (-0.752, 0.070)	0.025 (-0.021, 0.071)
Copper (mg)	2.37 (0.33)	0.047	-1.270	- 0.971, - 0.425	-0.643 (-0.829, - 0.167)	-0.027 (-0.197, 0.145)
Vitamin A (ER)	1492 (716)	0.109*	-869.425	- 0.938, - 0.068	-0.480 (-0.753, 0.068)	0.016 (-0.065, 0.095)
Thiamine (mg)	1.6 (3.1)	0.078*	-1.055	- 0.955, - 0.228	-0.535 (-0.779, - 0.003)	0.018 (-0.117, 0.448)
Riboflavin (mg)	0.07 (1.40)	0.078*	-1.615	- 0.955, - 0.228	-0.546 (-0.784, - 0.018)	0.033 (-0.052, 0.117)
Pantothenic acid (mg)	12.9 (3.1)	0.047	-6.712	- 0.971, - 0.425	-0.612 (-0.815, - 0.117)	0.040 (-0.031, 0.111)
Vitamin B6 (mg)	3.2 (1.3)	0.375*	-0.712	- 0.858, - 0.354	-0.286 (-0.657, 0.274)	0.129 (-0.136, 0.377)
Folic Acid (ugEFD)	1569 (1057)	1.000*	24.038	- 0.680, - 0.680	-0.098 (-0.553, 0.426)	-0.050 (-0.158, 0.058)

Niacin (mg)	13.5(0.30)	19.9(9.3)	0.297	-7.903	-21.930, 5.260
Vitamin C (mg)	146(11)	386(165)	0.05	-221.015	-439.710, -9.440

*Abbreviations: MAD median absolute deviation

* p value >0.05
