# Supplementary material for: Maternal Diet May Modulate Breast Milk Microbiota—A Case Study in a Group of Colombian Women
Source: Microorganisms. 2023 Jul 14;11(7):1812. doi: 10.3390/microorganisms11071812 (PMC10384792; doi:10.3390/microorganisms11071812)
Supplement: Supplementary file 1 [file microorganisms-11-01812-s001.zip › Table S2. Initial and post-filter sequencing reads.pdf]

Table S2. Initial and post-filter sequencing reads for each sample.

Sample identification	Initial sequences	Filtered	Filtered (%)	Denoised	Non chimeric sequences	Non chimeric sequences (%)
S1	132505	115167	86.92	114440	111059	83.81
S10	88478	78018	88.18	77710	73360	82.91
S11	96772	86938	89.84	86605	84984	87.82
S12	90956	80406	88.4	80080	78937	86.79
S13	95231	83730	87.92	83483	82623	86.76
S14	93092	82872	89.02	82716	80539	86.52
S15	95981	83814	87.32	83414	81473	84.88
S16	96570	86151	89.21	85600	83983	86.97
S17	79074	69036	87.31	68743	64698	81.82
S18	69513	60879	87.58	60621	56744	81.63
S19	82519	73540	89.12	73315	72651	88.04
S2	109188	92636	84.84	91662	90557	82.94
S20	115856	101708	87.79	100741	92978	80.25
S21	78828	70385	89.29	70162	65250	82.78
S22	77380	68429	88.43	68118	66231	85.59
S23	100264	87079	86.85	86834	84650	84.43
S24	84883	75601	89.06	75271	71042	83.69
S25	100468	88362	87.95	86641	63571	63.27
S26	87431	77808	88.99	76512	71773	82.09
S27	134239	118640	88.38	117806	97606	72.71
S28	80915	73143	90.39	72924	72067	89.07
S29	77609	68940	88.83	68703	67717	87.25
S3	100721	87969	87.34	87791	85589	84.98
S30	103062	91604	88.88	91385	88811	86.17
S4	95504	82726	86.62	82514	81447	85.28
S5	111428	98236	88.16	97731	96563	86.66
S6	123615	108096	87.45	107507	102610	83.01
S7	148449	131662	88.69	131266	126815	85.43
S8	110048	97630	88.72	97055	90997	82.69
S9	80397	69717	86.72	69586	69052	85.89
