# Supplementary figures and images for: Maternal Diet May Modulate Breast Milk Microbiota—A Case Study in a Group of Colombian Women
Source: Microorganisms. 2023 Jul 14;11(7):1812. doi: 10.3390/microorganisms11071812 (PMC10384792; doi:10.3390/microorganisms11071812)

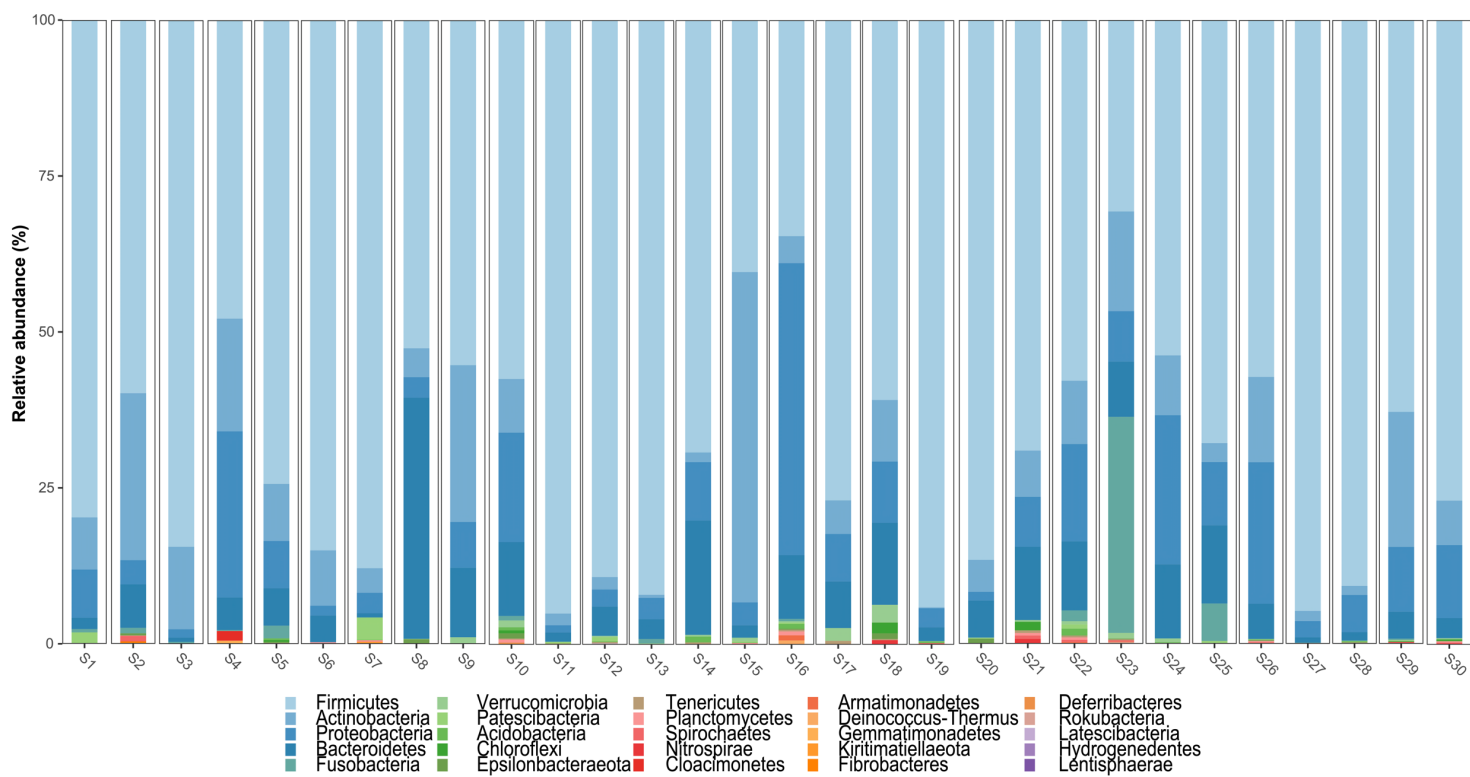

**Figure S1.** Bacterial phyla identified in breast milk microbiota.

Supplement: Supplementary file 1 [file microorganisms-11-01812-s001.zip › Figure S1. Bacterial phyla identified in breast milk microbiota..pdf]
